# Environmental Conditions Influence the Plant Functional Diversity Effect on Potential Denitrification

**DOI:** 10.1371/journal.pone.0016584

**Published:** 2011-02-02

**Authors:** Ariana E. Sutton-Grier, Justin P. Wright, Bonnie M. McGill, Curtis Richardson

**Affiliations:** 1 Nicholas School of the Environment and Earth Sciences, Duke University, Durham, North Carolina, United States of America; 2 Biology Department, Duke University, Durham, North Carolina, United States of America; Argonne National Laboratory, United States of America

## Abstract

Global biodiversity loss has prompted research on the relationship between species diversity and ecosystem functioning. Few studies have examined how plant diversity impacts belowground processes; even fewer have examined how varying resource levels can influence the effect of plant diversity on microbial activity. In a field experiment in a restored wetland, we examined the role of plant trait diversity (or functional diversity, (FD)) and its interactions with natural levels of variability of soil properties, on a microbial process, denitrification potential (DNP). We demonstrated that FD significantly affected microbial DNP through its interactions with soil conditions; increasing FD led to increased DNP but mainly at higher levels of soil resources. Our results suggest that the effect of species diversity on ecosystem functioning may depend on environmental factors such as resource availability. Future biodiversity experiments should examine how natural levels of environmental variability impact the importance of biodiversity to ecosystem functioning.

## Introduction

The loss of biodiversity due to human actions is a growing global concern [1], [2], [3]. Many studies have demonstrated that biodiversity affects the stability and functioning of ecosystems (see reviews in 4,5). Although there is general agreement that biodiversity can have a significant impact on plant productivity and plant resource consumption, [4], [5], [6] there continues to be some discussion over the mechanisms by which biodiversity impacts ecosystem function. In order to develop a mechanistic understanding of how biodiversity affects ecosystem function we must examine how the functional characteristics (i.e. traits) of organisms and functional diversity within a community relate to ecosystem function [7], [8].

Relatively few biodiversity studies have considered how plant biodiversity impacts belowground processes and microbial communities. As a result, there is a growing interest and need to determine the extent to which plant diversity might indirectly affect other ecosystem functions that are carried out by microbial communities [9], [10]. Microbial communities are structured by multiple environmental conditions, some of which are directly or indirectly altered by plants via their functional traits. Thus, the effect of biodiversity on microbial processes is likely dependent on interactions with environmental conditions [11]. Understanding the feedbacks between biodiversity, ecosystem functioning, and environmental factors is critical for understanding and predicting how changes in biodiversity will impact ecosystem processes [12]. Most biodiversity field studies to date have either ignored natural variability in soil variables or have manipulated resource levels to create large differences in available resource levels. In this study we explicitly examined the interaction between natural levels of variability in soil resources and plant functional diversity on a microbially-mediated ecosystem function, denitrification, in a restored riparian wetland.

**Figure 1:**
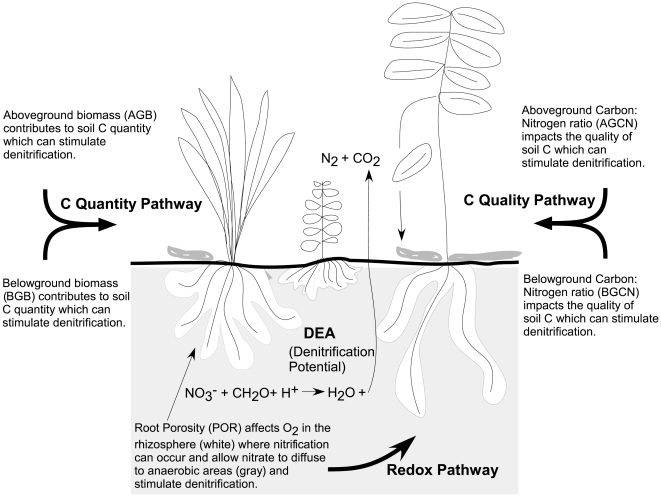
Conceptual diagram of how plant traits can influence denitrification potential (DNP). DNP is an ecosystem function that plants influence indirectly by modifying the soil environment. We hypothesized three categories of traits by which plant traits could impact DNP: (1) Carbon Quantity, (2) Carbon Quality, and (3) Redox (potential). We hypothesized that DNP will be promoted if the plant community trait values either increase (AGB, BGB, and POR) or decrease (AGCN and BGCN) such that C quantity, quality, and soil oxygen increase.

It is well established that soil conditions play an important role in driving denitrification. Both soil nitrogen (N) and soil carbon (C) are important drivers of denitrification rates [13], [14]. Soil moisture is also an important factor which drives the anaerobic conditions necessary for denitrification [15], [16]. Microbial biomass has also been shown to be positively correlated with denitrification potential rates [14]. The importance of these soil factors to denitrification suggests that plants may impact denitrification indirectly by influencing soil conditions which will in turn alter the microbial community. Other studies have found that specific types of plants, such as annuals, and specific species influence denitrification [17], [18], [19], [20], [21]. An observational field study determined that denitrification potential increased with increasing plant species richness [22], but a wetland mesocosm experiment did not find a relationship between plant functional group richness and denitrification potential [23]. Here, we specifically examined whether plant functional diversity influenced denitrification potential (see the section “Functional Diversity trait selection” in the methods section as well as Fig. 1 for a conceptual diagram of how plant traits influence soil conditions and denitrification potential). We chose to measure denitrification potential in the lab instead of denitrification in the field because field rates can exhibit high temporal variability since they are a product of the availability of denitrification substrates in the soil environment and not a measure of microbial community's functional potential. Additionally, denitrification potential measurements have been shown to give the clearest indication of differences in experimental treatments because denitrification potential measurements are sensitive to changes in factors that control denitrification such as soil C availability [24], [25]. Therefore, denitrification potential is likely the best way to measure the integrated effect of our treatments, different levels of plant FD, on the microbial community activity.

The effects of functional diversity and soil resources on denitrification are likely to be interactive and we can postulate multiple hypotheses about the nature of the interaction. On one hand, functional diversity may have the greatest impact at low resource levels, when conditions are the most stressful. In stressful environments, more diverse communities can display increased stability of plant production [26], [27] providing a buffering effect to ecosystem processes. On the other hand, functional diversity may have the largest impact at high resource levels, if plants are most productive under high nutrient levels [28], [29], and hence have the greatest impact on soil processes. A third possibility is that functional diversity will have a “hump-shaped” relationship with resource levels such that the biggest impact of functional diversity will be at intermediate levels of resources. This would be possible if at low levels of soil resources environmental conditions limit denitrification, and at high levels of available soil resources the benefits of functional diversity become irrelevant because resources are not limiting.

This study had two primary objectives. The first was to determine the relationship between plant trait diversity and a microbial process, denitrification potential. The second was to explicitly examine the nature of the interactions between plant trait diversity and environmental conditions making use of the range of soil resources present at our field site.

## Materials and Methods

### SWAMP study area and experimental design

Our experiment was located at the Duke University Stream and Wetland Assessment Management Park (SWAMP) along Sandy Creek in the Duke Forest in Durham, NC, USA. Soils in this area are primarily Cartecay silt loams and Mayodan sandy loams [30]. See Table 1 for a summary of soil properties at the site. In the process of restoration soils were homogenized as much as possible. Nevertheless, because soils are naturally heterogeneous, substantial variation in soil resources remained even after soils were graded and homogenized (See Table 1).

**Table 1:** Range of natural variability of soil variables at the Duke Forest field site. *

Soil Variable	Range
% Soil Moisture	18.21 – 30.04
% Soil Organic Matter	21.16–30.53
Inorganic Nitrogen (µg N g^−1^)	0.08–339.19
Microbial Biomass N (µg N g^−1^)	34.97–238.86

*See the “Soil sampling and laboratory analysis” section of the Materials and Methods section for a description of how these variables were measured.

One hundred 2×2 meter plots were planted in May 2005 with a total of 100 seedlings of 1, 4, or 8 species from a pool of 10 species. The species in the study (see list in Table 2) were selected from a list of recommended species for North Carolina stream restoration [31] based on commercial availability and to maximize trait diversity. Four monocultures for each species were planted except for two species (*Asclepias incarnata* and *Lobelia cardinalis*) which only had two monocultures planted. Thirty 4-species plots and thirty-two 8-species plots were planted with species randomly selected from the ten. Species within each plot were planted in equal densities and equally spaced but randomly placed within the plot.

**Table 2:** Trait values (Mean (SE)) for planted species and *Microstigium vimineum.*

Species	AGB (g)* +	BGB (g)* +	BGCN(n = 3)	AGCN* +	POR (%)(n = 3)
*Asclepias incarnata*	59 (9.0)	0.05 (0.003)	57.3 (3.26)	126.0 (25.9)	10 (2)
*Carex crinita*	229 (31.5)	0.42 (0.13)	44.5 (3.75)	51.3 (9.41)	17 (3)
*Carex lurida*	270 (6.4)	0.01 (0.05)	48.1 (6.19)	49.0 (13.2)	32 (3)
*Eupatorium fistulosum*	150 (82.4)	0.46 (0.21)	55.0 (3.37)	62.3 (10.4)	6 (4)
*Chasmanthium latifolium*	183 (46.4)	0.09 (0.04)	34.7 (3.74)	51.6 (8.03)	16 (1)
*Juncus effusus*	311 (94.2)	0.05 (0.02)	25.0 (0.68)	39.2 (5.34)	24 (4)
*Lobelia cardinalis*	32 (5.7)	0.07 (0.03)	22.4 (2.40)	45.6 (1.28)	2 (2)
*Microstegium vimineum*	70 (39.6)	0.13 (0.04)	54.0 (12.23)	57.8 (12.0)	3 (1)
*Panicum virgatum*	315 (10.55)	0.10 (0.04)	27.6 (9.45)	76.1 (1.43)	20 (3)
*Scirpus cyperinus*	219 (36.1)	0.04 (0.02)	32.9 (2.99)	36.9 (3.44)	30 (3)
*Vernonia noveboracensis*	225 (83.5)	0.16 (0.05)	55.5 (12.94)	54.7 (4.02)	9 (2)

* =  measured in the field plots; otherwise measured in the greenhouse;

+ =  n is variable depending on how many monocultures we had of the species (2, 3, or 4);

AGB  =  aboveground biomass, BGB  =  belowground biomass, BGCN  =  belowground C:N ratio (unitless), AGCN  =  aboveground C:N ratio (unitless), POR  =  root porosity (%).

### Functional Diversity trait selection

We chose to use plant trait diversity (i.e. functional diversity, FD) and not species richness or functional group richness as our metric of diversity, because we wanted to use a metric that explicitly measures functional traits that we could relate mechanistically to our hypotheses [32]. In selecting the traits to include in our calculation of FD, we recognized three pathways by which plants are likely to influence denitrification: C quality, C quantity, or the redox (or moisture) status of the soil (Fig. 1). Plants influence soil carbon quality and quantity, both of which have been shown to limit denitrification, via root exudates, root turnover, and aboveground litter inputs [14], [21], [33], [34], [35]. We selected two traits to measure the quality of C inputs: aboveground C:N ratio (AGCN) and belowground C:N (BGCN) ratio which represent the relative amount of energy in plant inputs to soils [36]. In terms of “C Quantity,” estimates suggest that between 0.5 and 5% of plant fixed C enters the rhizosphere through plants roots, but this flux of C into soils is regulated partly by the amount of root present, and partly by processes such as the amount of photosynthesis in the shoot [37]. As a result, we chose two measures of plant productivity, aboveground biomass (AGB) and belowground biomass (BGB), as indices of plant C inputs to soil. The ability of species to produce biomass in monoculture varies widely (Table 2) and this variation reflects important differences in their biology which should lead to differences in species' impacts on microbial communities.

A third pathway by which plants may affect denitrification is through modification of the redox conditions in the soil via root delivery of oxygen through radial oxygen loss. In an anaerobic wetland environment, root porosity can lead to oxygen release by roots, facilitating nitrification [38]. With increasing concentrations of soil nitrate, plants may take up more nitrate but microbes will also process more nitrate; available soil nitrate and rates of nitrification have both been found to be tightly related to denitrification [13], [35], [39]. Sutton-Grier and Megonigal [40] also determined that different plant species can have strong impacts on terminal electron acceptors in wetland soils via plant impacts on soil oxygen levels. Therefore, the plant trait we measured to examine plant effects on soil redox conditions was root porosity (POR). These five traits were used to calculate individual measures of plant trait diversity, FD, for each community. See Table 2 for details about each trait we selected.

### Functional Diversity trait measurements

We measured AGB, BGB, and AGCN in our field plots. AGB was calculated for each species as the average aboveground biomass harvested in September 2007 from the two field monocultures of each species; any invading species biomass was excluded from our calculation of AGB for each species monoculture. The one exception was *Microstegium vimineum*, an invasive grass that surrounded the restoration site, for which we did not have monocultures. For this species, we calculated aboveground biomass in several adjacent unplanted control plots with naturally recruited *M. vimineum* accounting for an average of 46% of the plot aboveground biomass. *M. vimineum* AGB was measured excluding any other species in the unplanted plots. We collected aboveground biomass in two 0.25 m^2^ quadrats that were bulked together from each plot. Samples were dried at 60°C for a week and then weighed to determine total biomass for each species. BGB was similarly calculated as the average total root biomass from the two soil cores (2.5 cm diameter) from the two monocultures of each species with the exception of *M. vimineum* for which we used the root biomass from the same unplanted control plots. We collected cores directly adjacent to the intended species in the monocultures in order to get the best estimate of root biomass for each species. AGCN ratios were measured on a FlashEA 1112 Elemental Analyzer (Thermo Scientific, Waltham, MA, USA.). See Table 2 for a list of trait values for each species.

Given the difficulty in ascertaining the species identity of below-ground material collected from the field, BGCN and root porosity were quantified by growing the 11 species in the greenhouse under conditions designed to replicate temperature, humidity, and photoperiod at the field site. We measured belowground C:N ratio (BGCN) also using a FlashEA 1112 Elemental Analyzer and root porosity (POR) using the pycnometer method [41].

### Functional Diversity calculation

We used the five measured traits (AGCN, BGCN, AGB, BGB and POR) to calculate functional diversity for denitrification using Petchey and Gaston's [42] metric FD. Traits were transformed into standard deviation units (z-scores) so that all traits would be equally weighted. These z-scores were used to calculate dendrograms with a calculated branch length for each species indicating how different each species was from the others. To calculate the final FD for each plot, the branch lengths for each species present in a plot were summed. Species were considered to be present in a plot when their biomass accounted for at least 10% of the total plot biomass. FD scores ranged from 3.24 to 9.64.

### Soil sampling and laboratory analysis

Soil samples were collected all at the same time in September 2007, the third growing season of the experiment. Samples were collected from all plots with healthy vegetation (95 of the original 100). Two soil samples (2.5 cm diameter) from each plot were collected from the upper 15 cm of each plot.

Upon arrival at the laboratory, the two cores collected from each plot were bulked together and sieved through a No. 4 (4.75 mm) sieve prior to analysis. All roots that would not pass through the sieve as well as roots that were easily removed by visual inspection were collected from each soil sample, briefly rinsed in DI to remove soil, dried at 60°C, and weighed to estimate BGB within each plot. A sub-sample of each soil was oven-dried at 105°C for 24 hours to determine the moisture content. This dried soil was then used to determine percentage soil organic matter (OM) by loss on ignition at 450°C [43]. Two replicate 3 g field-moist sub-samples were analyzed for 2 M KCl extractable nitrate + nitrite (NO_3_^−^ + NO_2_^−^) and ammonium (NH_4_^+^) [44] on a Lachat QuikChem 8000 (Lachat Instruments, Loveland, CO, USA.). The wetland soils at our site tended to be relatively oxic throughout most of the growing season which meant that soil N could be cycling between NO_3_ and NH_4_ at any one point in time. This cycling between N forms meant that the NO_3_ levels at any one sampling point might not fully represent the soil N available to the microbial community. Therefore, we chose to use total soil inorganic N (NO_3_ + NO_2_^−^ + NH_4_) because this was a better representation soil N levels at any one time [45].

Microbial biomass nitrogen (MB) was measured using four 5 g subsamples of wet soil and a slightly modified version of the Voroney and Winter [46] chloroform incubation technique. Chloroform (0.5 mL) was applied to cotton balls in the headspace of the fumigated sample containers and the samples incubated for 7 days in the dark before they were extracted with 0.5 M K_2_SO_4_. Non-fumigated samples were extracted immediately. Control and fumigated samples were analyzed for total nitrogen and MB was calculated for each sample as the difference between the fumigated and control values.

Denitrification Enzyme Assay (DEA) was used as a measure of denitrification potential using the standard method by Groffman et al. [25] which is based on Smith and Tiedje [47]. DEA measures potential denitrification because C and N are supplied in excess and the incubation is carried out under anaerobic conditions such that N_2_O gas produced is a function of the level of enzyme in the sample. In the lab, duplicate 5 g samples of homogenized, field-moist soil were weighed into 125 mL incubation flasks. Soil samples were amended with a solution of dextrose (0.5 g per L) and KNO_3_ (0.72 g per L) to ensure non-limiting substrate conditions and chloramphenicol (0.125 g per L) to inhibit protein synthesis. The slurries were made anaerobic with repeated flushing with N_2_ gas. Flasks were injected with 10 mL of acetylene to inhibit N_2_ production, making N_2_O the final product of denitrification. Flasks were placed on an orbital shaker and then gas samples were collected at 0, 30, 60, and 90 minutes and analyzed on a Shimadzu GC-17A gas chromatograph. Linear rates of accumulation of N_2_O were calculated.

### Statistical analyses

We used multiple linear regression to examine the importance of the environmental variables, FD, and their interactions in explaining DEA. Prior to analysis, microbial biomass N was log transformed to better conform to the assumptions of linear regression. We included all soil variables, FD, and all the interactions between soils variables and FD in our model and determined that all interactions were significant which meant we did not trim the model. To further explore the significant interactions, we used conditional plots, or coplots, which are a method for visualizing a significant interaction. Coplots enable one to graphically examine the relationship between two variables at differing levels of a third variable (for example, in this case we examined the relationship between plant FD and denitrification potential and differing levels of each environmental variable). In this way, coplots are an excellent tool to visually explore the nature of significant interactions between variables. However coplots represent a *qualitative* way to visualize statistically significant interactions; divisions of the data into different categories are performed mathematically so that there is the same number of data points in each category of the third variable. We experimented with dividing the data into two, three, or four levels of each environmental variable and the results were qualitatively similar. Thus, we chose to present the coplots using three divisions because it was the clearest way to represent the patterns in the interactions. Lowess, or locally-weighted scatterplot smoothing using least-squares, curves were fitted in the coplots with a span  = 0.9. While lowess curves provide a visual representation of the patterns in the data, significance of interactions was determined by the p-values in the multiple linear regression. Regression and coplot analyses were performed in the statistics package R 2.7.2 [48].

## Results

**Figure 2:**
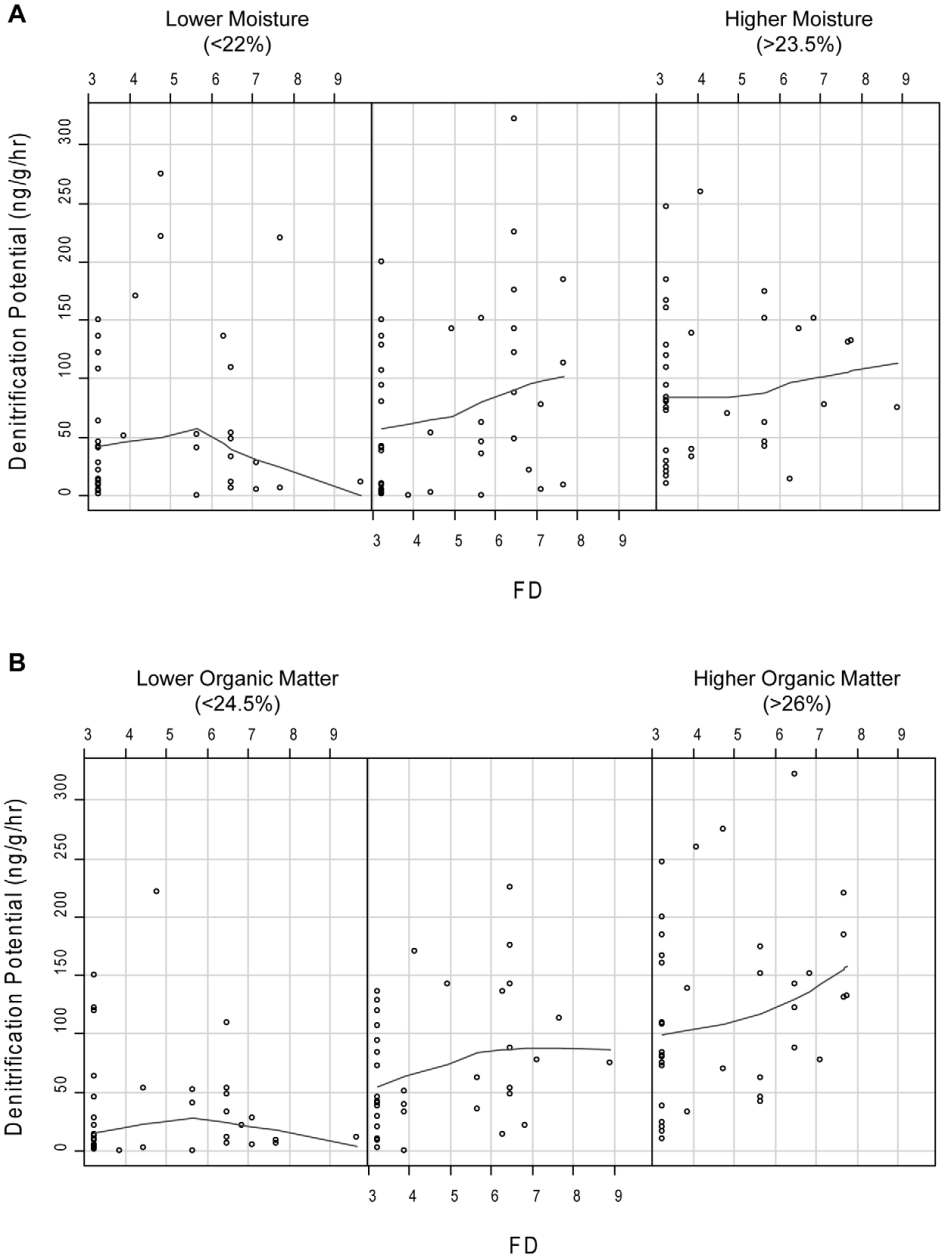
Coplots of denitrification potential (ng N g^−1^ hr^−1^) versus functional diversity (FD) conditional on the range of (A) soil % moisture and (B) soil % organic matter. The lines are the lowess (locally-weighted scatterplot smoothing using least-squares) curves that follow the trends in the data.

**Figure 3:**
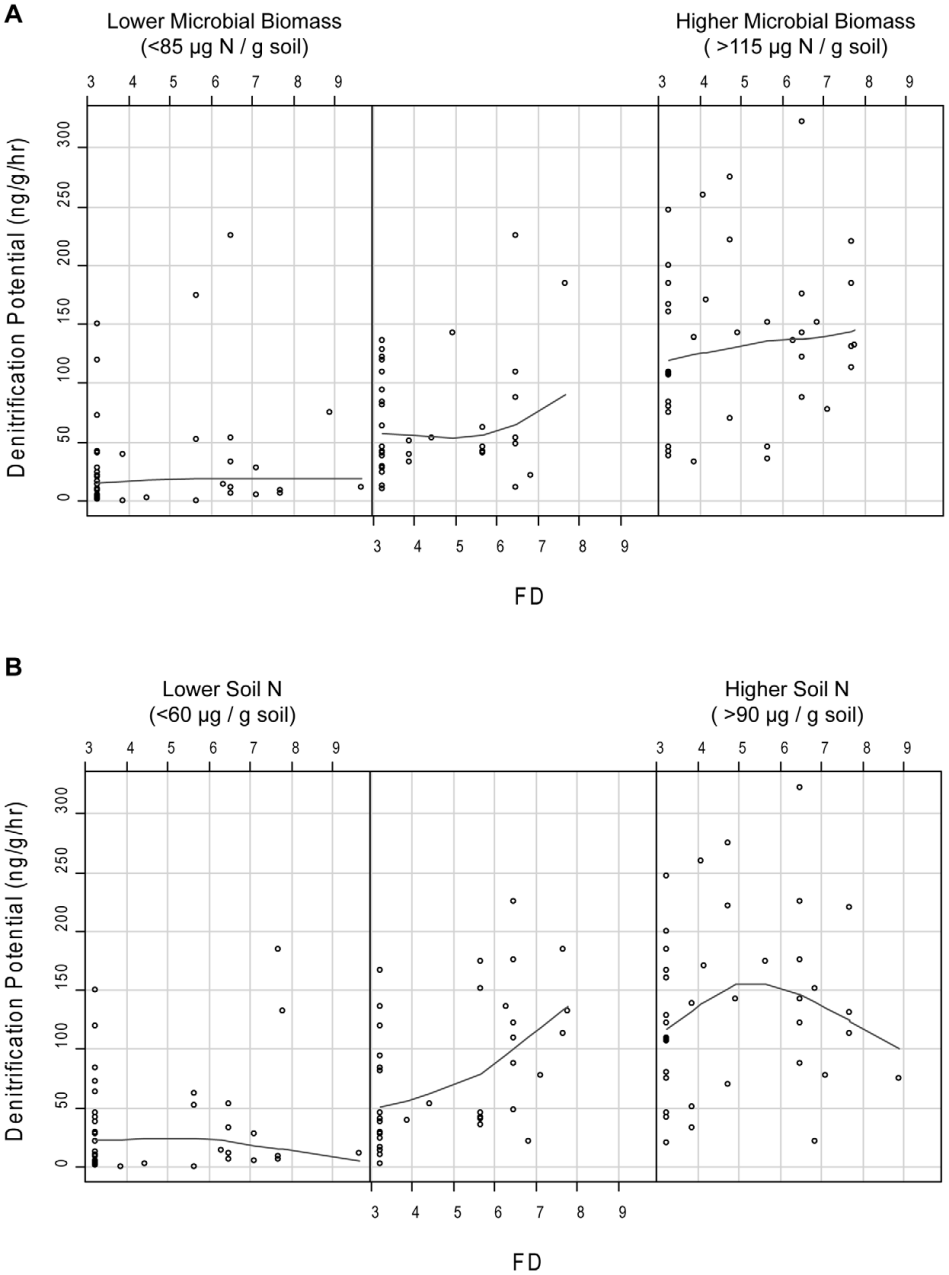
Coplots of denitrification potential versus functional diversity (FD) conditional on the range of (A) soil microbial biomass (µg N g^−1^ dry soil) and (B) soil inorganic N (µg g^−1^ dry soil). The lines are lowess (locally-weighted scatterplot smoothing) curves that follow the trends in the data.

When we included all the environmental variables, FD, and their interactions, stepwise analysis suggested all of the variables were important for explaining the variation in DNP. Therefore the final model included all environmental variables (% moisture, log microbial biomass N, % organic matter, and inorganic N), FD, and the interaction of FD with each environmental variable (Table 3). All soil properties had significant main effects on DNP while FD did not have a significant main effect. All the interactions of FD with each soil variable were significant, and three of the four interactions were highly significant (p<0.01, Table 3). This model explained 56% of the variability in DNP. Based on the visualization of these significant interactions with the coplots, it appears that there was a positive relationship between DNP and FD at higher levels of soil moisture, organic matter, and microbial biomass N (Figs. 2A, 2B and 3A). At lower levels of these three soil variables the relationship between DNP and FD tended to be nonexistent or slightly negative.

**Table 3:** Stepwise Multiple regression results to predict denitrification potential (Model R^2^ = 0.56).

Variable	Coefficient	Standard Error	t-statistic	P
Intercept	−573.63	242.23	−2.37	*
% Moisture	38.28	13.38	2.86	**
Log Microbial Biomass	280.81	69.22	4.06	***
% Organic Matter	−58.05	19.50	−2.98	**
Inorganic N	−0.93	0.45	−2.05	*
FD	58.46	49.99	1.17	n.s.
FD: % Moisture	−10.71	2.95	−3.64	***
FD: % Organic Matter	15.76	4.27	3.69	***
FD: Log Microbial Biomass	−49.24	15.06	−3.27	**
FD: Inorganic N	0.27	0.11	2.45	*

*P<0.05,

**P<0.01,

***P<0.001,

n.s.  = P>0.05.

The pattern in the interaction between DNP and FD at different levels of soil N was different based on the coplot visualization (Fig. 3B). At lower levels of soil N there appeared to be a slightly negative relationship, at mid-levels a strong positive relationship, and at higher levels a curvilinear relationship suggesting that the interaction between FD and soil N is more complicated than the other interactions.

## Discussion

### FD effects on DNP: Importance of environmental conditions

Our results indicate that plant functional diversity significantly influenced denitrification potential, but that the nature of the relationship depended on the available soil resources (Table 3). Some biodiversity studies have found that belowground processes do not respond as strongly to plant aboveground processes, such as aboveground biomass [49], [50]. But Zak et al. [9] suggested that plant-microbe interactions are an integral part of plant diversity's influence on ecosystem functions. We were particularly interested in determining whether plant diversity impacted denitrification, a microbial process, because of the variable results observed in previous studies looking at this question [22], [23]. Our results in this field study suggest that one of the reasons why other studies may have failed to find a consistent effect of plant diversity on belowground processes is that the impact of diversity may be dependent on other environmental factors such as soil conditions.

When we examined the effects of field-relevant, natural levels of variation in soil resources on the diversity effect, we found significant interactions between FD and soil moisture, OM, microbial biomass, and inorganic N. Therefore, it is important to examine the effect of FD in light of these interactions. Although some variability in soil resources may have been due to plant influences, there was also background variability in the soil resources at the site that remained throughout the experiment. This variability is evident when examining Figs. 2 and 3 because at each level of a particular soil resource, all levels of FD are present suggesting that the plant effects were imposed on top of a natural gradient in soil resources that was still present at the time of sampling.

We proposed three hypotheses for how FD could interact with soil conditions. Based on the visualization of the significant interactions using coplots, we found no support for our first hypothesis that FD might have the strongest impacts at low soil resource levels. In support of our second hypothesis, we found that for three of the four soils variables (soil moisture, organic matter, and microbial biomass) FD appeared to have the most positive effect at higher soil resource levels. This result is similar to other studies that have found stronger plant diversity effects at higher soil resource levels [28], [29], [51], [52], [53], [54]. However, these results are somewhat in contrast to the findings of Wacher et al. [55] who found an inconsistent net biodiversity effect; aboveground biomass increased with soil N fertilization treatment in some species mixtures, but decreased in others.

We also found some evidence to support our third hypothesis of a “hump-shaped” relationship between FD, environmental conditions, and denitrification. The effect of FD on denitrification appeared to peak at intermediate levels of soil N in our plots (at the low end of the higher soil N values in the third panel of Fig. 3B). This suggests that above a threshold value of soil N, plant functional diversity does not stimulate denitrification. At higher levels of soil N, it is likely that microbial denitrification may be limited by some other factor, such as C availability. If some other factor does become limiting at higher soil N levels, this would result in a hump-shape relationship with soil N.

Our study is one of the first to take a functional approach to looking at plant diversity (using plant trait diversity (FD)); previous studies have looked at species richness or plant functional group richness effects. Some studies have focused on how soil resource levels influence the diversity effect, however, these studies have examined plant species richness or functional group richness and have focused on plant productivity as the ecosystem function of interest, not microbial processes [28], [29], [51], [52], [53], [54]. There have been very few studies examining the general relationship between plant species diversity and soil microbial communities [9], and none examining the impact of plant FD on microbial communities. Chung et al. [56] found that plant species richness increased microbial and fungal biomass but only under treatments with either elevated CO_2_ or elevated soil N. Species richness had a much more variable influence on microbial enzyme activity demonstrating significant interactions with resource availability [56], results that are broadly consistent with our experiment. However, De Deyn et al. [57] did not find that soil fertility influenced the plant species richness or functional group richness effects on multiple ecosystem functions including stocks of C and N in vegetation, soils and soil microbes, or loss of C and N from soil through leaching.

### Future Research Directions

There were a few aspects of this study that suggest some future avenues for productive research. For example, due to the destructive nature of many trait measurements as well as limited space in the field, we were unable to measure all traits on individuals growing in the field. There may be some differences between traits measured on species in the greenhouse versus species grown in the field that we were unable to verify in this study. Currently, there is general uncertainty about the degree of phenotypic plasticity of traits important for ecosystem function. Several studies have demonstrated that abiotic factors, such as nutrient and water availability, and biotic factors, including the presence, absence, density, or identity of neighbors, influence plant phenotypic plasticity [See 58 for a good review of these studies]. But we know very little about the consequences of phenotypic plasticity on the interactions among plants and on plant community dynamics [58]. As a result, phenotypic plasticity, including in which species it occurs and under what conditions, is likely to be a very productive field for future research and will greatly help inform our understanding of how plant traits influence community dynamics including how biodiversity impacts ecosystem function.

Also, in addition to collecting data on microbial process rates, measurements of the microbial community, both structure and abundance, would provide a better mechanistic understanding of how changes in plant FD result in changes in the rate of a microbially-mediated process. Molecular microbial community data could suggest direct ways in which differences in plant FD affect microbial process rates. Although collecting molecular data was beyond the scope of this study, examining the links between changes in plant diversity, changes in microbial community structure, and how these changes affect microbial functioning is a key direction for future research [10].

In summary, we found that plant trait diversity had significant effects on the microbial soil process of denitrification through its interactions with soil conditions. Increasing FD led to increased DNP, primarily at higher levels of soil resources. These results, as well as other studies, suggest that future biodiversity experiments need to include a range of variable environments [29] or better account for the natural variability that occurs in ecosystems at the local scale. Future biodiversity experiments should be designed in order to examine how natural levels of environmental variability impact the importance of biodiversity to ecosystem functioning.
